# To the Reader

**Published:** 1883-12-20

**Authors:** 


### *TO THE READER.*

This number of our Journal will be sent to many who have, perhaps, not before seen it. It is not an average example of the practical character of the Journal and its adaptation to the wants of the busy practitioner.

If you will glance at the contents you will get an idea of the variety and wide scope of information contained in a volume of 480 pages of our Journal. Something may be seen on almost every topic in the field of practical medicine, and scarcely a single monthly issue can be found which does not, of itself, indemnify the subscriber for the small amount of $2.00 a year required as the subscription. Try it for six months at least, and you will not be likely soon to discontinue or to deprive yourself of the pleasure and benefit to be derived from a monthly visitor so useful and so valuable.

But lest it be said "Let another praise thee and not thyself," we give the following extract of a letter from Dr. P. F. Akers, of Georgia, which indeed is but one of a great many we have received equally complimentary. He writes:

"I like the size and plan of your Journal. It is just the thing for the busy practitioner. The terms are certainly very cheap for the evident care and judgment bestowed upon its contents, in which is a brief of the entire field of medical literature. The profession should sustain it in preference to the cheaper Journals, some of which are trashy, and others largely devoted to puffing the nostrums of the publishers. Such Journals are dear at any price. Were I confined to a single journal I would select yours in preference to any journal in the United States."

